# T-cell Cholesterol Accumulation, Aging, and Atherosclerosis

**DOI:** 10.1007/s11883-023-01125-y

**Published:** 2023-07-03

**Authors:** Venetia Bazioti, Benedek Halmos, Marit Westerterp

**Affiliations:** 1 Department of Pediatrics, University Medical Center Groningen, University of Groningen, Antonius Deusinglaan 1, Groningen, 9713AV The Netherlands; 2 Institute for Cardiovascular Prevention (IPEK), Ludwig-Maximilians-Universität, 80336 Munich, Germany

**Keywords:** T-cell, Atherosclerosis, ABC transporters, Cholesterol, CVD

## Abstract

**Purpose of Review:**

The majority of leukocytes in advanced human atherosclerotic plaques are T-cells. T-cell subsets exert pro- or anti-atherogenic effects largely via the cytokines they secrete. T_regulatory_ cells (T_regs_) are anti-inflammatory, but may lose these properties during atherosclerosis, proposed to be downstream of cholesterol accumulation. Aged T-cells also accumulate cholesterol. The effects of T-cell cholesterol accumulation on T-cell fate and atherosclerosis are not uniform.

**Recent findings:**

T-cell cholesterol accumulation enhances differentiation into pro-atherogenic cytotoxic T-cells and boosts their killing capacity, depending on the localization and extent of cholesterol accumulation. Excessive cholesterol accumulation induces T-cell exhaustion or T-cell apoptosis, the latter decreasing atherosclerosis but impairing T-cell functionality in terms of killing capacity and proliferation. This may explain the compromised T-cell functionality in aged T-cells and T-cells from CVD patients.

**Summary:**

The extent of T-cell cholesterol accumulation and its cellular localization determine T-cell fate and downstream effects on atherosclerosis and T-cell functionality.

## Introduction

T-cells make up ~50–65% of all leukocytes in advanced human atherosclerotic lesions from carotid endarterectomies [1••, 2••]. After infiltration into atherosclerotic lesions, T-cells interact with macrophages and dendritic cells (DCs) [3••]. Upon recognition of their cognate antigen presented by DCs and dependent on the cytokine milieu, naïve T-cells differentiate into distinct subsets characterized by the expression of transcription factors (i.e., FoxP3 for T regulatory cells (T_regs_); T_bet_ for T helper 1 (T_h_1) cells; GATA3 for T_h_2 cells; retinoic acid-related orphan receptor (ROR)γT for T_h_17 cells; and B-cell lymphoma (Bcl)6 for T_follicular helper_ (T_fh_) cells) [3••]. More than 80% of T-cells in atherosclerotic plaques express CD44, indicating that they are antigen experienced [2••, 3••]. Among the antigens that DCs present to T-cells are apolipoprotein B100 (apoB100), low-density lipoprotein (LDL), and oxidized LDL [4–6]. While initial studies have suggested that antigen presentation in atherosclerotic plaques induces production of the pro-atherogenic T_h_1 cytokines interferon γ (IFNγ) and tumor necrosis factor α (TNFα) [4, 7], later studies have shown an expansion of T_regs_ in response to antigens [6, 8, 9]. T_regs_ exert an anti-atherogenic role by secreting interleukin (IL)-10 and transforming growth factor β (TGF-β) [10, 11]. TGF-β induces smooth muscle cell (SMC) migration and collagen production by SMCs [12–15]. Recent single-cell RNA sequencing (sc-RNA-Seq) studies have revealed a high diversity of T-cells in human atherosclerotic plaques [1••, 2••]. The role of specific T-cell subsets in atherosclerosis has been reviewed previously [3••].

Even though individual T-cell subsets have pro- or anti-atherogenic effects, presumably via the cytokines they secrete, complete CD4^+^ or CD8^+^ T-cell ablation reduces atherosclerosis in mice [16–19]; however, in advanced atherosclerosis, CD8^+^ T-cell ablation increases plaque stability [20•], highlighting the complex role of T-cells in atherogenesis.

Recent studies have revealed that during atherosclerosis and cardiovascular disease (CVD) in humans, T_regs_ acquire markers of T_h_1, T_h_17, and T_fh_ cells, or switch to a more memory-like phenotype, which may render them pro-atherogenic [6, 9, 21, 22, 23•, 24]. Studies in mouse models have proposed that T-cell cholesterol accumulation critically contributes to this effect [23•]. In addition, T-cells from CVD patients lose their ability to proliferate and, therefore, to respond adequately to antigens [25•], a critical function of T-cells. The decreased proliferation may be the result of T-cell apoptosis downstream of excessive T-cell cholesterol accumulation [26••]. Aged T-cells also show increased cholesterol accumulation [27, 28]. Here, we will review how pathways that regulate T-cell cholesterol accumulation determine T-cell fate, atherosclerosis, and T-cell aging.

## T-cell Receptor Stimulation and Cholesterol Accumulation

**Fig. 1:**
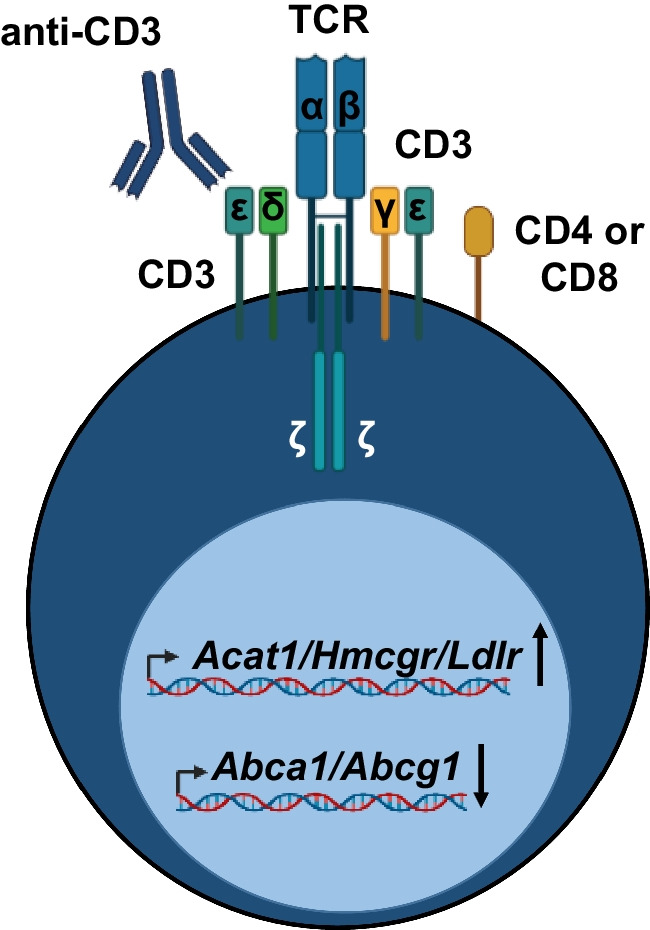
Effects of anti-CD3 stimulation on expression of genes involved in cholesterol homeostasis. Gene transcription is shown in the nucleus. Created with BioRender.com

T-cells express high levels of the cholesterol transporters ATP Binding Cassette A1 (ABCA1) and ABCG1 that mediate cholesterol efflux to apolipoprotein A-I (apoA-I) and high-density lipoprotein (HDL), respectively [29]. T-cells mainly accumulate cholesterol in their plasma membrane, which is key to T-cell receptor (TCR) signaling and proliferation in response to interaction with their cognate antigen. TCR stimulation by anti-CD3, which mimics T-cell stimulation by antigen-presenting cells via major histocompatibility complex (MHC)I/II, decreases expression of the cholesterol transporters *Abca1* and *Abcg1* (Fig. 1) [30••, 31••]. The decreased expression of ABC cholesterol transporters is mediated by suppression of Liver X receptor (LXR) signaling due to upregulation of the enzyme sulfotransferase family cytosolic 2B member 1 (SULT2B1) that transfers sulfate groups to oxysterols, which inactivates oxysterols in terms of their ability to bind the transcription factor LXR and to activate it [30••, 32]. TCR stimulation also increases the expression of 3-hydroxy-3-methylglutaryl-CoA reductase (*Hmgcr*), the LDL receptor (*Ldlr*), and acetyl coA acyl transferase 1 (*Acat1*), which promote cholesterol synthesis, uptake, and esterification, respectively (Fig. 1) [30••, 31••].

## T-cell Membrane Cholesterol Accumulation Induces T-cell Proliferation

Several lines of evidence indicate that cholesterol accumulation is key to T-cell proliferation and, as such, key to the T-cell response upon interaction with an antigen. Suppression of cholesterol synthesis due to deficiency of sterol regulatory element-binding protein (SREBP) cleavage-activating protein (SCAP) completely abolishes T-cell proliferation in response to anti-CD3 [33•]. Conversely, when cholesterol cannot be esterified due to deficiency of *Acat1*, plasma membrane cholesterol accumulation increases, as does T-cell proliferation [31••]. Similarly, deficiency of *Abcg1*-mediated cholesterol efflux promotes plasma membrane cholesterol accumulation and T-cell proliferation [30••, 34, 35]. T-cell cholesterol loading via methyl-β-cyclodextrin (MβCD)-cholesterol or LDL-cholesterol (LDL-c) also increases proliferation [34, 36].

*Abcg1* deficient T-cells show high expression of *Abca1* [34], presumably due to the accumulation of oxysterols that induce the activation of LXR and consequently *Abca1* transcription [37,38]. Recent studies have revealed that T-cell *Abca1* deficiency increases *Abcg1* expression, reduces T-cell membrane cholesterol accumulation, and decreases T-cell proliferation in response to anti-CD3 [39]. These data suggest that, as initially proposed [30••], *Abcg1* is the dominant cholesterol transporter in T-cells. We found that deficiency of both *Abca1* and *Abcg1* increases T-cell membrane cholesterol accumulation and proliferation in young mice [26••]. Conversely, incubation with reconstituted HDL (rHDL) that induces cholesterol efflux, shows the opposite [26••]. Recent studies revealed that *histone deacetylase 3* (*Hdac3*) deficiency decreases T-cell proliferation, which was attributed to decreased membrane cholesterol accumulation and increased *Abca1* and *Abcg1* mRNA expression [40•]. These data substantiate the crucial role for cholesterol efflux pathways in regulating T-cell proliferation. An overview of pathways regulating cholesterol accumulation and T-cell proliferation is given in Table 1.

**Table 1:** Plasma membrane cholesterol and T-cell proliferation

Model	Plasma membrane cholesterol	T-cell proliferation	T-cell subtypes
*Lxrβ* deficiency [30••]	Not reported	↑	Total T-cells
*Abcg1* deficiency [30••, 34, 35]	↑	↑	CD4^+^
T-cell *Abca1* deficiency [39]	↓	↓	CD4^+^ or CD8^+^
T-cell *Acat1* deficiency [31••]	↑	↑	CD8^+^
T-cell *Scap* deficiency [33•]	↓	↓	CD8^+^
T-cell *Hdac3* deficiency [40•]	↓	↓	CD4^+^
MβCD-cholesterol (20 μg/mL; 2 hours prior to TCR stimulus) [34]	Not reported	↑	CD4^+^
LDL-c (72 hours during TCR stimulus) [36]	Not reported	↑	CD8^+^
T-cell *Abca1/Abcg1* deficiency, young [26••]	↑	↑	CD4^+^ or CD8^+^
rHDL (50 μg/mL; 72 hours during TCR stimulus) [26••]	Not reported	↓	CD4^+^ or CD8^+^

T-cells were stimulated with αCD3/αCD28 for 72 h to induce proliferation; except for [34] (66 h)

Abca1 and Abcg1, ATP binding cassette A1 and G1; *Acat1*, acyl-CoA cholesterol acyltransferase 1; *Hdac3*, histone deacetylase 3; *LDL*, low-density lipoprotein; *Lxrβ*, liver X receptor β; *MβCD*-cholesterol, methyl-β-cyclodextrin cholesterol; *rHDL*, reconstituted high-density lipoprotein; *Scap*, sterol regulatory element-binding protein (SREBP) cleavage-activating protein; *TCR*, T-cell receptor

## T-cell Proliferation During Aging and CVD

While combined T-cell *Abca1/Abcg1* deficiency increased T-cell proliferation in young mice, T-cell *Abca1/Abcg1* deficiency almost abolished T-cell proliferation in mice at 1 year of age, concomitant with an upregulation of the senescence marker p21 [26••]. These findings suggest that perhaps aged *Abca1/Abcg1* deficient T-cells became senescent due to several rounds of homeostatic proliferation. In addition, *Abca1/Abcg1* deficiency increased T-cell apoptosis, in both young mice and mice at 1 year of age [26••]. The increase in T-cell apoptosis may be more prominent during aging, as such contributing to the abolished T-cell proliferation in aged mice.

Interestingly, individuals over 70 years of age also show T-cell cholesterol accumulation compared to T-cells from individuals less than 25 years of age [27, 28], as do T-cells from wild-type mice at 2 years of age compared to T-cells from wild-type mice at 3 months of age [26••]. T-cells from aged mice (2 years) show increased apoptosis compared to T-cells from young mice (3 months) [26••]. Based on the findings in mice with T-cell *Abca1/Abcg1* deficiency [26••], these data suggest that also during aging, T-cell cholesterol accumulation contributes to apoptosis and, consequently, the decline in total T-cells. T-cell proliferation was only minimally decreased in T-cells from aged mice compared to young mice [26••]. However, T-cells from *Apolipoprotein e* deficient (*Apoe*^*−/−*^) mice with advanced atherosclerosis due to 20 weeks of cholesterol-rich Western-type diet (WTD) feeding, show decreased T-cell proliferation and increased T-cell apoptosis compared to T-cells from *Apoe*^*−/−*^ mice fed a chow diet [25•]. Even though this was attributed to impaired antigen presentation by DCs [25•], previous studies have shown that WTD feeding induces cholesterol accumulation in *Apoe*^*−/−*^ T-cells [23•], and our studies in mice with T-cell *Abca1/Abcg1* deficiency demonstrate that T-cell cholesterol accumulation may directly increase T-cell apoptosis [26••].

In line with the findings in *Apoe*^*−/−*^ mice, patients with advanced coronary artery disease (CAD) show a decrease in proliferation and an increase in T-cell apoptosis compared to patients with early CAD, irrespective of age (*n* = 14 patients per group) [25•]. While this would need to be confirmed in a larger CAD cohort, the data suggest a direct link between advanced CAD and impaired T-cell functionality due to T-cell apoptosis. Our data show that T-cell cholesterol accumulation, which may be aggravated in advanced CAD, contributes to this impaired T-cell functionality.

Not all genes that affect T-cell membrane cholesterol accumulation and TCR signaling (Table 1) affect apoptosis. *Acat1* deficiency decreased apoptosis in CD8^+^ T-cells [31••], perhaps due to *Acat1* deficiency increasing T-cell proliferation and survival, which may offset potential effects on apoptosis. However, it should be noted that the effects of *Abca1/Abcg1* or *Apoe* were most pronounced in CD4^+^ T-cells [25•, 26••], and are probably the consequence of an increase in intracellular T-cell membrane cholesterol accumulation that is more dramatic than reported for other genes listed in Table 1. Nonetheless, T-cell cholesterol accumulation induced by MβCD-cholesterol loading promotes endoplasmic reticulum (ER) stress and CD8^+^ T-cell exhaustion without affecting apoptosis [41•]. We found that also in aged T-cells from wild-type mice (2 years old), expression of SREBP2 was decreased compared to T-cells from young mice (3 months), suggestive of ER cholesterol accumulation [26••]. ER cholesterol accumulation may account for T-cell exhaustion during aging.

## T-cell Membrane Cholesterol Accumulation and Differentiation into Cytotoxic T-cells

In addition to T-cell proliferation, TCR stimulation increases granzyme B, IFNγ, and TNFα positive CD8^+^ T-cells, which are required for killing of foreign cells or pathogens [42]. Similar to effects on T-cell proliferation, deletion of genes or treatments that favor cholesterol accumulation (*Lxrβ* deficiency [30••], *Acat1* deficiency [31••], MβCD-cholesterol [31••], and LDL-c [36]) induce differentiation into these cytotoxic CD8^+^ T-cells, while a decrease in cholesterol synthesis by *Scap* deficiency [33•] or treatment with lovastatin [31••] or cholesterol depletion by MβCD [31••] does the opposite. Also, inhibition of Niemann-Pick C1 protein, which induces movement of cholesterol from lysosomes to the plasma membrane, by the U18666A compound, decreases differentiation into these cytotoxic T-cells [31••], presumably due to decreased plasma membrane cholesterol [43]. These findings are summarized in Table 2. In line, T-cell *Abca1/Abcg1* deficiency induces differentiation into granzyme B and IFNγ expressing CD8^+^ T-cells [26••]. However, T-cell *Abca1/Abcg1* deficiency decreased IFNγ secretion and T-cell mediated macrophage killing [26••]. We attributed these effects to increased T-cell apoptosis, and therefore these effects are simply the consequence of a lower number of T-cells [26••]. Similarly, T-cells from *Apoe*^*−/−*^ mice fed WTD for 20 weeks show decreased IFNγ production compared to *Apoe*^*−/−*^ mice fed a chow diet, concomitant with increased apoptosis [25•].

**Table 2:** Plasma membrane cholesterol and granzyme B^+^, IFNγ^+^, and TNFα^+^ CD8^+^ T-cells

Model	Time prior to TCR stimulus	Plasma membrane cholesterol	Granzyme B^+^ CD8^+^	IFNγ^+^ CD8^+^	TNFα^+^ CD8^+^
*Lxrβ* deficiency [30••]	-	Not reported	Not reported	↑	↑
T-cell *Acat1* deficiency [31••]	-	↑	↑	↑	↑
MβCD-cholesterol (10 μg/mL) [31••]	15 minutes	↑	↑	↑	↑
MβCD (1mM) [31••]	5 minutes	↓	↓	↓	↓
T-cell *Scap* deficiency [33•]	-	↓	Not reported	↓	↓
LDL-c (24 hours during TCR stimulus) [36]	-	Not reported	↑	↑	↑
Lovastatin (10 μM) [31••]	6 hours	Not reported	↓	↓	↓
U18666A (2 μg/mL) [31••]	6 hours	Not reported	↓	↓	↓
T-cell *Abca1/Abcg1* deficiency [26••]	-	↑	↑	↑	Not reported

T-cells were stimulated with αCD3/αCD28 for 24 h; except for T-cell *Abca1/Abcg1* deficiency where the stimulus was αCD3/IL-2 for 12 h. For *Lxrβ* deficiency and T-cell *Scap* deficiency, T-cells were stimulated by immunization in vivo

Abca1 and Abcg1, ATP binding cassette A1 and G1; *Acat1*, acyl-CoA cholesterol acyltransferase 1; *IFNγ*, interferon γ; *IL-2*, interleukin 2; *LDL*, low-density lipoprotein; *Lxrβ*, liver X receptor β; *MβCD*, methyl-β-cyclodextrin; *Scap*, sterol regulatory element-binding protein (SREBP) cleavage-activating protein; *TCR*, T-cell receptor; *TNFα*, tumor necrosis factor α

## T-cell Membrane Cholesterol Accumulation, Atherosclerosis, and CVD

While effects of membrane cholesterol accumulation on T-cell proliferation and differentiation into cytotoxic T-cells seem to be relatively uniform, effects of T-cell cholesterol accumulation on downstream T-cell differentiation are not. T-cell *Abcg1* deficiency increases membrane cholesterol accumulation and lipid droplet formation, indicative of increased cholesterol esterification [35]. T-cell *Abcg1* deficiency increases formation of T_regs_, with athero-protective effects [35].

**Fig. 2:**
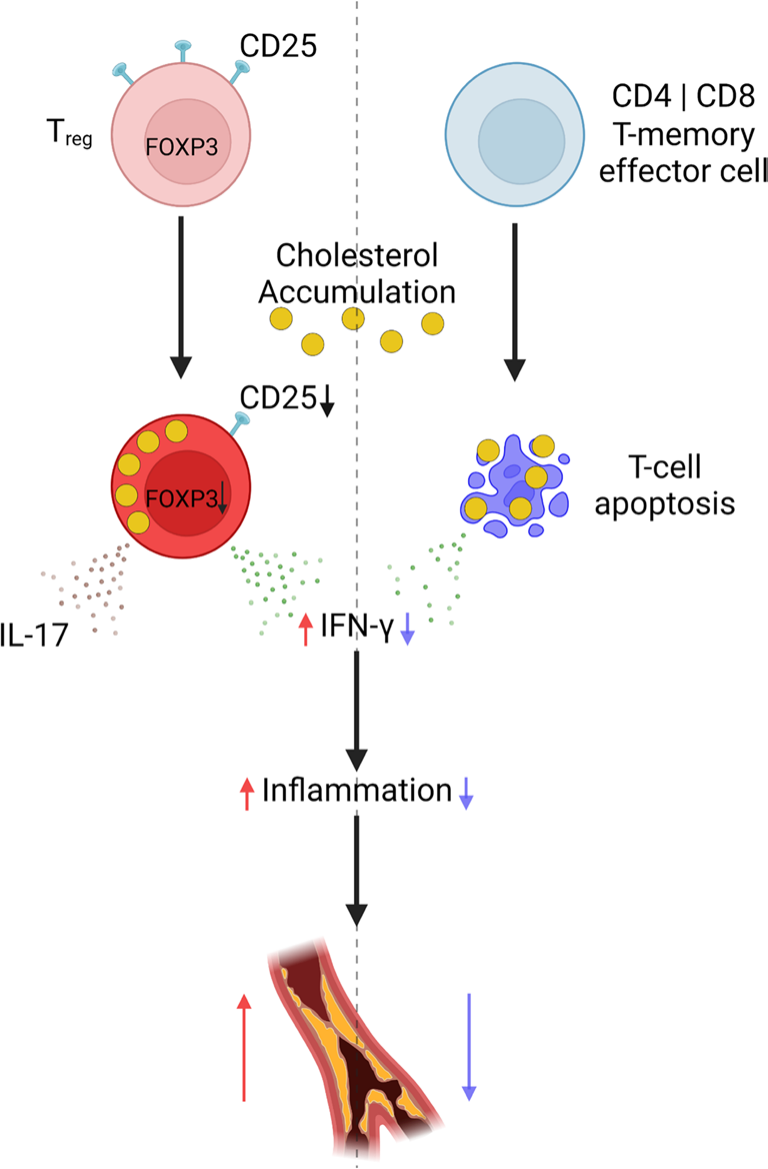
Effects of cholesterol accumulation on regulatory T-cell (T_reg_) fate and on T-cell apoptosis and downstream effects on the production of interferon γ (IFNγ), inflammation, and atherosclerosis. Created with BioRender.com

Recent studies have revealed that during atherosclerosis and CVD, T_regs_ acquire markers of T_h_1, T_h_17, and T_fh_ cells, which may render them pro-atherogenic [6, 9, 21, 22, 23•, 24] (Fig. 2). Using a fluorescent tracing technique, current T_regs_ and exT_regs_ (cells that were T_regs_ before) could be distinguished in *Apoe*^*−/−*^ mice [23•]. This revealed that upon WTD feeding, T_regs_ underwent a phenotypic switch [23•]. Injections of apoA-I reversed this switch [23•], and therefore this switch was proposed to occur downstream of cholesterol efflux and thus to be cholesterol-dependent. In this model, T_regs_ lost their Foxp3 and CD25 expression and started to express IFNγ or Bcl6 and IL-21, suggesting differentiation into T_h_1 or T_fh_ cells, respectively [23•]. Previous sc-RNA-Seq studies have indeed shown that T_regs_ gain features of T_h_1 cells during atherosclerosis in *Apoe*^*−/−*^ mice and that these cells are dysfunctional in terms of suppressing T-cell proliferation, a main characteristic of T_regs_ [22]. Deficiency of the specific T_fh_ transcription factor *Bcl6* decreased atherosclerosis, indicating that T_fh_ cells are pro-atherogenic [23•], presumably because they induce B-cell activation and secretion of IL-21 [44,45]. One caveat to this atherosclerosis study was that Bcl6 is also expressed by germinal center B-cells that have a pro-atherogenic role [44,46]. Nonetheless, this study [23•] strongly suggests that cholesterol accumulation in T_regs_ compromises T_reg_ function and enhances atherogenesis. This outcome is different from the mice with T-cell *Abcg1* deficiency that showed cholesterol accumulation and increased T_regs_. This may be due to a higher level of membrane cholesterol accumulation in T-cells from *Apoe*^*−/−*^ mice fed a WTD than in WTD-fed *Ldlr*^*−/−*^ mice with T-cell *Abcg1* deficiency, simply because in the setting of T-cell *Abcg1* deficiency cholesterol esters accumulate [35], which may not have been the case in *Apoe*^*−/−*^ mice fed WTD.

Interestingly, mice with T-cell *Abca1* deficiency show a decrease in T_regs_ [47], attributed to increased *Abcg1* expression [39], but T-cell *Abca1* deficiency is athero-protective in *Ldlr*^*−/−*^ mice fed WTD [39]. This athero-protective effect was attributed to a decrease in membrane cholesterol accumulation due to elevated *Abcg1* expression, and a decrease in T_memory effector_ cells that indeed may have a pro-atherogenic role [39]. In contrast, we recently found that combined T-cell *Abca1/Abcg1* deficiency decreased T_memory effector_ cells but did not affect atherosclerosis in young *Ldlr*^*−/−*^ mice fed WTD, while decreasing atherosclerotic plaque size in *Ldlr*^*−/−*^ mice fed a chow diet at 1 year of age [26••]. We attributed the latter to the higher number of T-cells in plaques of *Ldlr*^*−/−*^ mice at 1 year of age than in young mice, and thus a more prominent role of T-cells in plaque formation in aged mice [26••]. Mechanistically, T-cell *Abca1/Abcg1* deficiency increased T-cell apoptosis and, consequently, decreased IFNγ production, decreasing macrophage inflammation in lesions [26••] (Fig. 2). Even though *Apoe*^*−/−*^ mice also show decreased T-cell IFNγ production after 20 weeks of WTD feeding, this does not compromise lesion growth [25•], presumably because pro-inflammatory effects of *Apoe* deficiency on other cell types, such as macrophages, are dominant. *Apoe*^*−/−*^ mice fed WTD may resemble advanced CAD in humans [25•], and therefore these studies in *Apoe*^*−/−*^ mice are most informative in providing mechanistic insights as to why T-cells in patients with advanced CAD lose their funtionality in terms of proliferation and IFNγ production, likely occurring downstream of increased T-cell apoptosis.

## Conclusions and Future Directions

T-cell membrane cholesterol accumulation is key to T-cell proliferation and differentiation into cytotoxic T-cells both processes downstream of TCR signaling that are crucial to T-cell function [30••, 33•]. The exact mechanism for these findings is not yet clear. Membrane cholesterol accumulation may induce TCR clustering [31••], as such activating TCR signaling; however, studies employing artificial membranes have yielded conflicting data as to the role of membrane cholesterol in TCR signaling [48, 49], indicating that the exact mechanism remains to be elucidated.

During aging, T-cell numbers decline, and T-cells accumulate cholesterol [27, 28]. Cholesterol accumulation may induce T-cell apoptosis or T-cell exhaustion [26••, 41•], which both may contribute to the decrease in T-cell numbers.

The diminished T-cell functionality in terms of T-cell proliferation and IFNγ production in CAD patients may be the consequence of T-cell cholesterol accumulation [25•, 26••]. Similarly, cholesterol accumulation in T_regs_ of CVD patients may enhance differentiation into pro-atherogenic T-cell subsets [6, 9, 21, 22, 23•, 24] (Fig. 2). Although deficiency of T-cell cholesterol efflux pathways also increased T-cell apoptosis in atherosclerotic plaques [26••], it seems rather unlikely that high levels of cholesterol accumulation in T-cells from human atherosclerotic plaques have a similar effect. Even though the plaque environment is rich in cholesterol, plaques from human carotid endarterectomies show high numbers of T-cells [1••, 2••, 50••] that differentiate into specific T-cell subsets completely dependent on the local plaque environment [50••]. Triggers that regulate this differentiation remain to be determined. Recent single TCR sequencing studies suggest that atherosclerosis has an auto-immune component driven by autoreactive CD4^+^ T-cells [50••].

In conclusion, several findings, as summarized in Tables 1 and 2, indicate that T-cell membrane cholesterol accumulation is key to regulating the functionality of peripheral T-cells. This is particularly important in response to infections. Indeed, a lack of cholesterol synthesis in CD8^+^ T-cells resulted in an attenuated clonal T-cell expansion during viral infection [33•]. Excessive cholesterol accumulation compromises T-cell functionality by inducing T-cell apoptosis [26••]. This may contribute to the increase in T-cell apoptosis and impaired T-cell functionality in patients with advanced CAD [25•].

## References

[1] Depuydt MAC, Prange KHM, Slenders L, Ord T, Elbersen D, Boltjes A, de Jager SCA, Asselbergs FW, de Borst GJ, Aavik E, Lonnberg T (2020). Microanatomy of the human atherosclerotic plaque by single-cell transcriptomics. Circ Res..

[2] Fernandez DM, Rahman AH, Fernandez NF, Chudnovskiy A, Amir ED, Amadori L, Khan NS, Wong CK, Shamailova R, Hill CA, Wang Z (2019). Single-cell immune landscape of human atherosclerotic plaques. Nat Med..

[3] Saigusa R, Winkels H, Ley K (2020). T cell subsets and functions in atherosclerosis. Nat Rev Cardiol..

[4] Stemme S, Faber B, Holm J, Wiklund O, Witztum JL, Hansson GK (1995). T lymphocytes from human atherosclerotic plaques recognize oxidized low density lipoprotein. Proc Natl Acad Sci United States Am..

[5] Hermansson A, Ketelhuth DF, Strodthoff D, Wurm M, Hansson EM, Nicoletti A, Paulsson-Berne G, Hansson GK (2010). Inhibition of t cell response to native low-density lipoprotein reduces atherosclerosis. J Exp Med..

[6] Kimura T, Kobiyama K, Winkels H, Tse K, Miller J, Vassallo M, Wolf D, Ryden C, Orecchioni M, Dileepan T, Jenkins MK (2018). Regulatory cd4(+) t cells recognize major histocompatibility complex class ii molecule-restricted peptide epitopes of apolipoprotein b. Circulation.

[7] Koltsova EK, Garcia Z, Chodaczek G, Landau M, McArdle S, Scott SR, von Vietinghoff S, Galkina E, Miller YI, Acton ST, Ley K (2012). Dynamic t cell-apc interactions sustain chronic inflammation in atherosclerosis. J Clin Investig..

[8] van Puijvelde GH, Hauer AD, de Vos P, van den Heuvel R, van Herwijnen MJ, van der Zee R, van Eden W, van Berkel TJ, Kuiper J (2006). Induction of oral tolerance to oxidized low-density lipoprotein ameliorates atherosclerosis. Circulation.

[9] Wolf D, Gerhardt T, Winkels H, Michel NA, Pramod AB, Ghosheh Y, Brunel S, Buscher K, Miller J, McArdle S, Baas L (2020). Pathogenic autoimmunity in atherosclerosis evolves from initially protective apolipoprotein b(100)-reactive cd4(+) t-regulatory cells. Circulation.

[10] Ait-Oufella H, Salomon BL, Potteaux S, Robertson AK, Gourdy P, Zoll J, Merval R, Esposito B, Cohen JL, Fisson S, Flavell RA (2006). Natural regulatory t cells control the development of atherosclerosis in mice. Nat Med..

[11] Klingenberg R, Gerdes N, Badeau RM, Gistera A, Strodthoff D, Ketelhuth DF, Lundberg AM, Rudling M, Nilsson SK, Olivecrona G, Zoller S (2013). Depletion of foxp3+ regulatory t cells promotes hypercholesterolemia and atherosclerosis. J Clin Investig..

[12] Doran AC, Meller N, McNamara CA (2008). Role of smooth muscle cells in the initiation and early progression of atherosclerosis. Arterioscler Thromb Vasc Biol..

[13] Mallat Z, Gojova A, Marchiol-Fournigault C, Esposito B, Kamate C, Merval R, Fradelizi D, Tedgui A (2001). Inhibition of transforming growth factor-beta signaling accelerates atherosclerosis and induces an unstable plaque phenotype in mice. Circ Res..

[14] Lutgens E, Gijbels M, Smook M, Heeringa P, Gotwals P, Koteliansky VE, Daemen MJ (2002). Transforming growth factor-beta mediates balance between inflammation and fibrosis during plaque progression. Arterioscler Thromb Vasc Biol..

[15] Amento EP, Ehsani N, Palmer H, Libby P (1991). Cytokines and growth factors positively and negatively regulate interstitial collagen gene expression in human vascular smooth muscle cells. Arterioscler Thromb..

[16] Emeson EE, Shen ML, Bell CG, Qureshi A (1996). Inhibition of atherosclerosis in cd4 t-cell-ablated and nude (nu/nu) c57bl/6 hyperlipidemic mice. Am J Pathol..

[17] Zhou X, Robertson AK, Rudling M, Parini P, Hansson GK (2005). Lesion development and response to immunization reveal a complex role for cd4 in atherosclerosis. Circ Res..

[18] Kyaw T, Winship A, Tay C, Kanellakis P, Hosseini H, Cao A, Li P, Tipping P, Bobik A, Toh BH (2013). Cytotoxic and proinflammatory cd8+ t lymphocytes promote development of vulnerable atherosclerotic plaques in apoe-deficient mice. Circulation.

[19] Cochain C, Koch M, Chaudhari SM, Busch M, Pelisek J, Boon L, Zernecke A (2015). Cd8+ t cells regulate monopoiesis and circulating ly6c-high monocyte levels in atherosclerosis in mice. Circ Res..

[20] van Duijn J, Kritikou E, Benne N, van der Heijden T, van Puijvelde GH, Kroner MJ, Schaftenaar FH, Foks AC, Wezel A, Smeets H, Yagita H (2019). Cd8+ t-cells contribute to lesion stabilization in advanced atherosclerosis by limiting macrophage content and cd4+ t-cell responses. Cardiovasc Res..

[21] Li J, McArdle S, Gholami A, Kimura T, Wolf D, Gerhardt T, Miller J, Weber C, Ley K (2016). Ccr5+t-bet+foxp3+ effector cd4 t cells drive atherosclerosis. Circ Res..

[22] Butcher MJ, Filipowicz AR, Waseem TC, McGary CM, Crow KJ, Magilnick N, Boldin M, Lundberg PS, Galkina EV (2016). Atherosclerosis-driven treg plasticity results in formation of a dysfunctional subset of plastic ifngamma+ th1/tregs. Circ Res..

[23] Gaddis DE, Padgett LE, Wu R, McSkimming C, Romines V, Taylor AM, McNamara CA, Kronenberg M, Crotty S, Thomas MJ, Sorci-Thomas MG (2018). Apolipoprotein ai prevents regulatory to follicular helper t cell switching during atherosclerosis. Nat Commun..

[24] Saigusa R, Roy P, Freuchet A, Gulati R, Ghosheh Y, Suthahar SSA, Durant CP, Hanna DB, Kiosses WB, Orecchioni M, Wen L (2022). Single cell transcriptomics and tcr reconstruction reveal cd4 t cell response to mhc-ii-restricted apob epitope in human cardiovascular disease. Nat Cardiovasc Res..

[25] Gaddis DE, Padgett LE, Wu R, Nguyen A, Mc Skimming C, Dinh HQ, Araujo DJ, Taylor AM, CA MN, Hedrick CC (2021). Atherosclerosis impairs naive cd4 t-cell responses via disruption of glycolysis. Arterioscler Thromb Vasc Biol..

[26] Bazioti V, La Rose AM, Maassen S, Bianchi F, de Boer R, Halmos B, Dabral D, Guilbaud E, Flohr-Svendsen A, Groenen AG, Marmolejo-Garza A (2022). T cell cholesterol efflux suppresses apoptosis and senescence and increases atherosclerosis in middle aged mice. Nat Commun..

[27] Larbi A, Dupuis G, Khalil A, Douziech N, Fortin C, Fulop T (2006). Differential role of lipid rafts in the functions of cd4+ and cd8+ human t lymphocytes with aging. Cell Signal..

[28] Larbi A, Fortin C, Dupuis G, Berrougui H, Khalil A, Fulop T (2014). Immunomodulatory role of high-density lipoproteins: impact on immunosenescence. Age (Dordr).

[29] Groenen AG, Halmos B, Tall AR, Westerterp M (2021). Cholesterol efflux pathways, inflammation, and atherosclerosis. Crit Rev Biochem Mol Biol..

[30] Bensinger SJ, Bradley MN, Joseph SB, Zelcer N, Janssen EM, Hausner MA, Shih R, Parks JS, Edwards PA, Jamieson BD, Tontonoz P (2008). Lxr signaling couples sterol metabolism to proliferation in the acquired immune response. Cell.

[31] Yang W, Bai Y, Xiong Y, Zhang J, Chen S, Zheng X, Meng X, Li L, Wang J, Xu C, Yan C (2016). Potentiating the antitumour response of cd8(+) t cells by modulating cholesterol metabolism. Nature.

[32] Chen W, Chen G, Head DL, Mangelsdorf DJ, Russell DW (2007). Enzymatic reduction of oxysterols impairs lxr signaling in cultured cells and the livers of mice. Cell Metab..

[33] Kidani Y, Elsaesser H, Hock MB, Vergnes L, Williams KJ, Argus JP, Marbois BN, Komisopoulou E, Wilson EB, Osborne TF, Graeber TG (2013). Sterol regulatory element-binding proteins are essential for the metabolic programming of effector t cells and adaptive immunity. Nat Immunol..

[34] Armstrong AJ, Gebre AK, Parks JS, Hedrick CC (2010). Atp-binding cassette transporter g1 negatively regulates thymocyte and peripheral lymphocyte proliferation. J Immunol..

[35] Cheng HY, Gaddis DE, Wu R, McSkimming C, Haynes LD, Taylor AM, McNamara CA, Sorci-Thomas M, Hedrick CC (2016). Loss of abcg1 influences regulatory t cell differentiation and atherosclerosis. J Clin Investig..

[36] Yuan J, Cai T, Zheng X, Ren Y, Qi J, Lu X, Chen H, Lin H, Chen Z, Liu M, He S (2021). Potentiating cd8(+) t cell antitumor activity by inhibiting pcsk9 to promote ldlr-mediated tcr recycling and signaling. Protein Cell..

[37] Janowski BA, Willy PJ, Devi TR, Falck JR, Mangelsdorf DJ (1996). An oxysterol signalling pathway mediated by the nuclear receptor lxr alpha. Nature.

[38] Yvan-Charvet L, Ranalletta M, Wang N, Han S, Terasaka N, Li R, Welch C, Tall AR (2007). Combined deficiency of abca1 and abcg1 promotes foam cell accumulation and accelerates atherosclerosis in mice. J Clin Investig..

[39] Zhao Y, Zhang L, Liu L, Zhou X, Ding F, Yang Y, Du S, Wang H, Van Eck M, Wang J (2022). Specific loss of abca1 (atp-binding cassette transporter a1) suppresses tcr (t-cell receptor) signaling and provides protection against atherosclerosis. Arterioscler Thromb Vasc Biol..

[40] Wilfahrt D, Philips RL, Lama J, Kizerwetter M, Shapiro MJ, SA MC, Kennedy MM, Rajcula MJ, Zeng H, Shapiro VS (2021). Histone deacetylase 3 represses cholesterol efflux during cd4(+) t-cell activation. eLife.

[41] Ma X, Bi E, Lu Y, Su P, Huang C, Liu L, Wang Q, Yang M, Kalady MF, Qian J, Zhang A (2019). Cholesterol induces cd8(+) t cell exhaustion in the tumor microenvironment. Cell Metab..

[42] Zhang N, Bevan MJ (2011). Cd8(+) t cells: Foot soldiers of the immune system. Immunity.

[43] Zhang JR, Coleman T, Langmade SJ, Scherrer DE, Lane L, Lanier MH, Feng C, Sands MS, Schaffer JE, Semenkovich CF, Ory DS (2008). Niemann-pick c1 protects against atherosclerosis in mice via regulation of macrophage intracellular cholesterol trafficking. J Clin Investig..

[44] Ait-Oufella H, Herbin O, Bouaziz JD, Binder CJ, Uyttenhove C, Laurans L, Taleb S, Van Vre E, Esposito B, Vilar J, Sirvent J (2010). B cell depletion reduces the development of atherosclerosis in mice. J Exp Med..

[45] Ding R, Gao W, He Z, Liao M, Wu F, Zou S, Ma L, Liang C, Wu Z (2014). Effect of serum interleukin 21 on the development of coronary artery disease. APMIS.

[46] Kitano M, Moriyama S, Ando Y, Hikida M, Mori Y, Kurosaki T, Okada T (2011). Bcl6 protein expression shapes pre-germinal center b cell dynamics and follicular helper t cell heterogeneity. Immunity.

[47] Gaddis DE, Wu R, Parks JS, Sorci-Thomas MG, Hedrick CC Lack of apolipoprotein a1 impairs optimal regulatory t cell homeostasis at steady state due to impaired il-2 signaling. BioRxiv (2019).

[48] Molnar E, Swamy M, Holzer M, Beck-Garcia K, Worch R, Thiele C, Guigas G, Boye K, Luescher IF, Schwille P, Schubert R (2012). Cholesterol and sphingomyelin drive ligand-independent t-cell antigen receptor nanoclustering. J Biol Chem..

[49] Swamy M, Beck-Garcia K, Beck-Garcia E, Hartl FA, Morath A, Yousefi OS, Dopfer EP, Molnar E, Schulze AK, Blanco R, Borroto A (2016). A cholesterol-based allostery model of t cell receptor phosphorylation. Immunity.

[50] MAC D, Schaftenaar FH, KHM P, Boltjes A, Hemme E, Delfos L, de Mol J, de Jong MJM, MNA BK, JAHM P, Goncalves L (2023). Single-cell t cell receptor sequencing of paired human atherosclerotic plaques and blood reveals autoimmune-like features of expanded effector t cells. Nat Cardiovasc Res..

